# Two new Species of *Pristionchus* (Nematoda: Diplogastridae) include the Gonochoristic Sister Species of *P. fissidentatus*

**DOI:** 10.21307/jofnem-2019-024

**Published:** 2019-04-24

**Authors:** Matthias Herrmann, Natsumi Kanzaki, Christian Weiler, Kohta Yoshida, Christian RÖdelsperger, Ralf J. Sommer

**Affiliations:** 1 Department of Evolutionary Biology, Spemannstraße 37, Max Planck Institute for Developmental Biology, Tübingen, Germany; 2 Kansai Research Center, Forestry and Forest Products Research Institute, Kyoto 612-0855, Japan

**Keywords:** *Pristionchus pacificus*, *P. fissidentatus*, Taiwan, Japan, Scarab beetles

## Abstract

The genus *Pristionchus* (Kreis, 1932) consists of more than 30 soil nematode species that are often found in association with scarab beetles. Three major radiations have resulted in the “*maupasi* species group” in America, the “*pacificus* species group” in Asia, and the “*lheritieri* species group,” which contains species from Europe and Asia. Phylogenetic analysis indicates that a group of three species, including the gonochorists *P. elegans* and *P. bucculentus* and the hermaphrodite *P. fissidentatus*, is basal to the above-mentioned radiations. Two novel species are described here: *Pristionchus paulseni* sp. n. from Taiwan and *P. yamagatae* sp. n. from Japan by means of morphology, morphometrics and genome-wide transcriptome sequence analysis. Previous phylotranscriptomic analysis of the complete *Pristionchus* genus recognized *P. paulseni* sp. n. as the sister species of *P. fissidentatus*, and thus its importance for macro-evolutionary studies. Specifically, the gonochorist *P. paulseni* sp. n. and the hermaphrodite *P. fissidentatus* form a species pair that is the sister group to all other described *Pristionchus* species. *P. paulseni* sp. n. has two distinct mouth forms, supporting the notion that the mouth dimorphism is ancestral in the genus *Pristionchus*.

The genus *Pristionchus* (Kreis, 1932) with the model organism *P. pacificus* has emerged as an important group of nematodes to study various aspects of macroevolution, including comparative developmental biology, phenotypic plasticity, evolutionary ecology, and comparative genomics (Sommer et al., 1996; Sommer, 2015). Many of these studies critically depend on deep taxon sampling and a robust phylogenetic framework (Susoy et al., 2015). The isolation of new *Pristionchus* species was fostered by the original discovery that these nematodes are often found in a necromenic and entomophilic association with scarab beetles (Herrmann et al., 2006, 2007; Kanzaki et al., 2012b). Subsequent systematic samplings resulted in the characterization of more than 30 *Pristionchus* species with four distinct biogeographically restricted species groups (Ragsdale et al., 2015; Herrmann et al., 2016).

The three major radiations include the “*maupasi* species group” in America, the “*pacificus* species group” in Asia and the “*lheritieri* species group,” which contains species from Europe and Asia (Ragsdale et al., 2015). As outgroup to these radiations, the “*elegans* species group” found in Asia consists of only two gonochorists: *P. elegans* and *P. bucculentus* (Kanzaki et al., 2012a; Ragsdale et al., 2013, 2015). However, an additional hermaphroditic species, *P. fissidentatus,* is an outgroup to the “*elegans* species group” and is the sister group to all described beetle-associated *Pristionchus* species. Given that most of the seven hermaphroditic species in the genus have evolved independently of one another, they allow the detailed analysis of parallel evolution, e.g., the influence of mating-type transitions on longevity (Weadick and Sommer, 2016; Rödelsperger et al., 2018). *P. fissidentatus* is of particular interest, given its phylogenetic position; however, it is a hermaphroditic species that has no sister group relationship with any particular species.

Here, we describe two new species. *P. yamagatae* sp. n. was isolated from *Holotricha sp.* (Coleoptera: Melolonthinae) in Japan and belongs to the *lheritieri*-species-complex. *P. paulseni* sp. n. was isolated from Taiwan and represents the gonochoristic sister species of *P. fissidentatus*. This species exhibits a mouth dimorphism found in many *Pristionchus* species, suggesting that mouth-form plasticity is an ancestral character in the genus. Most importantly, *P. paulseni* sp. n. and *P. fissidentatus* form a new species group of the genus that represents the sister group to all described beetle-associated *Pristionchus* species. Thus, the gonochorist *P. paulseni* sp. n. has a crucial position in the phylogeny of *Pristionchus*. In order to characterize both species, morphology, morphometrics, mating experiments and genome-wide transcriptome sequencing are used.

## Materials and methods

### Nematode cultivation

One strain of *P. paulseni* sp. n. was isolated from an adult of *Lucanus dybowski taiwanus* (Miwa, 1937) (Coleoptera: Lucanidae) collected at Pilu sacred tree, Taroko National Park, Taiwan. The strain of *P. yamagatae* sp. n. was isolated from an adult of *Holotrichia sp.* (Hope, 1837) (Coleoptera: Scarabaeidae, Melolonthinae) collected at Yuza, Yamagata, Japan. Both strains have been kept in laboratory culture on NGM agar plates seeded with *Escherichia coli* strain OP50 under the culture code and freezing voucher numbers RS5918 (*P. paulseni* sp. n.) and RS5964 (*P. yamagatae* sp. n.).

### Morphological observation and preparation of type material

Light microscopic observations for drawings and morphometrics were conducted using live nematode material, which was handpicked from culture plates (Kanzaki et al., 2013).

### Molecular characterization and phylogenetic analysis

A species phylogeny of the complete *Pristionchus* genus was reconstructed as described in Rödelsperger et al. (2018). In the following, we give a brief summary about the generation of the *Pristionchus* phylogeny (please see Rödelsperger et al., (2018) for full details). For all cultivable *Pristionchus* species, worms were grown on NGM plates seeded with *E. coli* OP50 at 20°C and total RNA was isolated from 2–3 mixed-stage plates per species using standard Trizol extraction following the manufacturers’ instructions (Zymo Research, CA, USA). RNA-seq libraries were prepared using TruSeq RNA library preparation kit v2 (Illumina, Inc., CA, USA), according to the manufacturer’s instructions, from 1 µg of total RNA in each sample and sequenced on the Illumina HiSeq3000 platform, yielding a median of 14m paired reads (2 × 150 bp) per species. Raw reads were submitted to the European nucleotide archive under the study accession PRJEB20959. RNA-seq reads were assembled into transcriptomes using Trinity (version v2.2.0) (Grabherr et al., 2011). For further analysis, only the first reported isoform per gene was selected and the longest complete or partial ORF (⩾60 amino acids) was called. Orthologous clusters were generated by orthAgogue (Ekseth et al., 2014) and protein sequences were aligned using the MUSCLE software (version 3.8.31) (Edgar, 2004). 2,092 high quality alignments containing at least 14 species (without any duplication), with at least 50 amino acid positions with coverage in all represented species, were concatenated into a supermatrix spanning 350,000 amino acids. On the basis of the previous analysis of dozens of gene families (Baskaran et al., 2015), we chose the LG substitution model to reconstruct a maximum-likelihood tree using RA × ML (version 8.2.9, options –m PROTGAMMAILG –f a –N 100) (Stamatakis, 2014).

## Results

### Description of common characters

The majority of *Pristionchus* species are cryptic and typologically similar to each other, including the two novel species described here. In general, *Pristionchus* species are distinguished from each other by stomatal and male tail characters only (for review see Ragsdale et al., 2015). Therefore, the general morphology, e.g., body shape and gonadal structures, is described first as common characters, and then the distinctive characters are described for each species.

### Adult

Their characteristics include a cylindrical and stout body, a thick cuticle, with fine annulation and clear longitudinal striations. Lateral field consists of two lines, weakly distinguishable from body striation with the presence of deirid. Head exists without apparent lips and with six short and papilliform labial sensillae. Four small, papilliform cephalic papillae are present in males, as typical for diplogastrid nematodes. Amphidial apertures are located on the lateral sector, slightly dorsally shifted, at level of margin of cheilo- and gymnostom. Stomatal dimorphism is present, with stenostomatous (narrow mouthed) and eurystomatous (wide mouthed) forms occurring in both males and females, but male eurystomatous form is not as common as females. Detailed stomatal morphology is described below. Dorsal pharyngeal gland is clearly observed, with a penetrating dorsal tooth to gland opening. The anterior part of pharynx (=pro and metacorpus) is 1.5 times as long as the posterior part (=isthmus and basal bulb). Procorpus is very muscular, stout, occupying half to two-thirds of the corresponding body diameter. Metacorpus is very muscular, forming a well-developed median bulb. Isthmus is narrow, not muscular. Basal bulb is glandular. Pharyngo-intestinal junction is clearly observed; it is well-developed. Nerve ring is usually surrounding the middle region of isthmus. Excretory pore is not conspicuous, ventrally located at the level of isthmus to pharyngo-intestinal junction; excretory duct extends anteriad and is reflexed back to the position of pore. Deirid is observed laterally, located from the level of pharyngo-intestinal junction to one body diameter and posterior to the junction. Hemizonid is not clearly observed. Lateral glands, small pores connected to secretory cell, are present and observed on the lateral body surface, with positions inconsistent among individuals, numbering 5 to 8 for males and 9 to 13 for females. Because the structure is very small and indistinctive, the consistency of their position and number was not confirmed by light microscopy.

### Stenostomatous form

Cheilostom consists of six per- and interradial plates. Incision between plates is not easily distinguished by light microscopy. The anterior end of each plate is usually rounded and elongated to project from stomatal opening and forms a small flap. Some more details are described for each species below. The anterior end of each plate sometimes splits into two, forming two narrow flaps, i.e., number of cheilostomatal flaps varies from 6 to 12, regardless of the number of plates (=6). Gymnostom is short, with a cuticular ring-like anterior end overlapping cheilostom internally. Dorsal gymnostomatal wall is slightly thickened compared to the ventral side. Stegostom is divided into three subsections: pro-meso, meta and telostegostom. Pro-meso stegostom forms a weak cuticularized ring surrounding the anterior edge of pharynx. Metastegostom bears a conspicuous and movable triangular or flint-shaped dorsal tooth with strongly sclerotized surface, giving an appearance of an inverted V-shape in light microscopy in lateral view; a pointed left subventral ridge with three minute adventitious denticles on a plate is present; a pointed right subventral ridge exists, often with distinct distal adventitious denticle(s). Telostegostom forms a weakly sclerotized cup-like cavity connecting stoma and pharynx.

### Eurystomatous form

Cheilostom is divided into six distinctive per- and interradial plates. The anterior end of each plate is usually rounded and elongated to project from stomatal opening, forming a small flap. The anterior part of each plate is sometimes split into two, forming two narrow flaps, i.e., number of cheilostomatal flaps varies from 6 to 12, regardless of the number of plates (=6). Gymnostom is present with a thick cuticle, forming a short, ring-like tube being thicker posteriorly. The anterior end of gymnostom is internally overlapping the posterior end of cheilostomatal plates. Stegostom is divided into three subsections: pro-meso, meta and telostegostom. Pro-mesostegostom forms a weak cuticularized ring surrounding the anterior edge of pharynx. Metastegostom bears large claw-like dorsal tooth, large claw-like right subventral tooth, ridge of left subventral denticles or cusps, varying in number and size. Dorsal and right subventral teeth are movable. No movement is observed for left subventral denticles. Telostegostom forms a weak sclerotized cup-like cavity connecting stoma and pharynx.

### Male

They are ventrally arcuate and become strongly ventrally curved at tail region when killed by heat. Testis is single and ventrally located; the anterior part is reflexed to the right side. Spermatogonia is arranged in three to five rows in the reflexed part; then well-developed spermatocytes are arranged in three to four rows in anterior two-thirds of the main branch; then mature amoeboid spermatids are arranged in multiple rows in remaining, proximal part of gonad. *Vas deferens* is not clearly separated from other parts of gonad. Three (two subventral and one dorsal) cloacal gland cells are observed at distal ends of testis and intestine. Spicules are paired and separate. Spicules are smoothly curved in ventral view, adjacent to each other for distal third of their length, smoothly tapering to pointed distal end. Spicule in lateral view is smoothly ventrally arcuate, giving spicule about 100° curvature; oval manubrium is present at the anterior end; lamina/calomus complex (blade) is clearly expanded slightly posterior to manubrium (*ca* 1/4 of blade length from anterior), smoothly tapering to pointed distal end. Gubernaculum conspicuous, about one-third of spicule length, is broad anteriorly such that dorsal wall is slightly recurved and that dorsal and ventral walls separate at 50 to 60° angle at the posterior end. The dorsal side of gubernaculum possesses a single, membranous, anteriorly directed process and a lateral pair of more sclerotized, anteriorly and obliquely ventrally directed processes. In lateral view, anterior half of gubernaculum is present with two serial curves separated by anteriorly and obliquely ventrally directed process, with anterior terminal curvature being highly concave and almost closed, and with deep posterior curvature being one-third of the gubernaculum length; posterior half forms a tube-like process enveloping spicules. A thick cuticle exists around tail region, falsely appearing as a narrow leptoderan bursa in ventral view. Cloacal opening (co) is slit-like in ventral view. One small, ventral, single genital papilla (vs) exists on anterior cloacal lip. Nine pairs of genital papillae (v1-v7, ad, pd) and a pair of phasmids (ph) are present, with three precloacal and six postcloacal pairs. Within those pairs, three distal ventral pairs (v5, v6, and v7) and a dorsal pair (pd) are close to each other around the posterior end of tail (just anterior to the root of spike). Anterior five pairs of papillae (v1-4 and ad) are present, almost equal in size, rather large and conspicuous; v7 and pd papillae are obviously smaller than anterior five pairs; v5 and v6 are very small, sometimes difficult to observe with light microscope. Anterior two pairs of the ventral triplet papillae (v5 and v6) papilliform are present and born from a socket-like base, v7 is simple or typical thorn-like in shape. The tip of v6 papillae is split into two small papilla-like projections. A detailed arrangement of papillae and phasmid will be described for each species below. The tail is conical, with a long spike about two to three cloacal body diameter. Bursa or bursal flap is absent.

### Female

They are relaxed or slightly ventrally arcuate when killed by heat. Gonad is didelphic, amphidelphic. Each gonadal system is arranged from vulva/vagina as uterus, oviduct, and ovary. The anterior gonad is in right of intestine, with uterus and oviduct extending ventrally and anteriorly on right of intestine and with a totally reflexed (=antidromous reflexion) ovary extending dorsally on left of intestine. Oocytes are mostly arranged in three to four rows in distal two-thirds of ovary and in double or single row in rest of the ovary, with distal tips of each ovary reaching oviduct of the opposite gonad branch. The anterior end of oviduct (= junction tissue between ovary and oviduct) consists of rounded cells. The anterior part of oviduct consists of rounded cells forming simple tube. The middle part of oviduct serves as spermatheca and consists of roundish and relatively large cells. Eggs in single to multiple-cell stage or even further stages are developed at the posterior part of oviduct (= uterus), which in young female is composed of squared or angular cells, long enough to contain one well-developed oocyte. *Receptaculum seminis* is not observed, *i.e.*, the organ is not independent and is a part of oviduct/uterus. Vaginal glands are present but obscure. Vagina is perpendicular to the body surface, surrounded by sclerotized tissue. Vulva is slightly protuberant in lateral view and pore-like in ventral view. Rectum is about one anal body diameter long, intestine/rectum junction is surrounded by a well-developed sphincter muscle. Three anal glands (two subventral and one dorsal) are present but not obvious. Anus is in the form of dome-shaped slit, posterior anal lip slightly protuberant. Phasmid is about one to two anal body diameter long, posterior to anus. Tail is long, smoothly tapered to distal end variable from filiform to long and conical.

**Figure 1:**
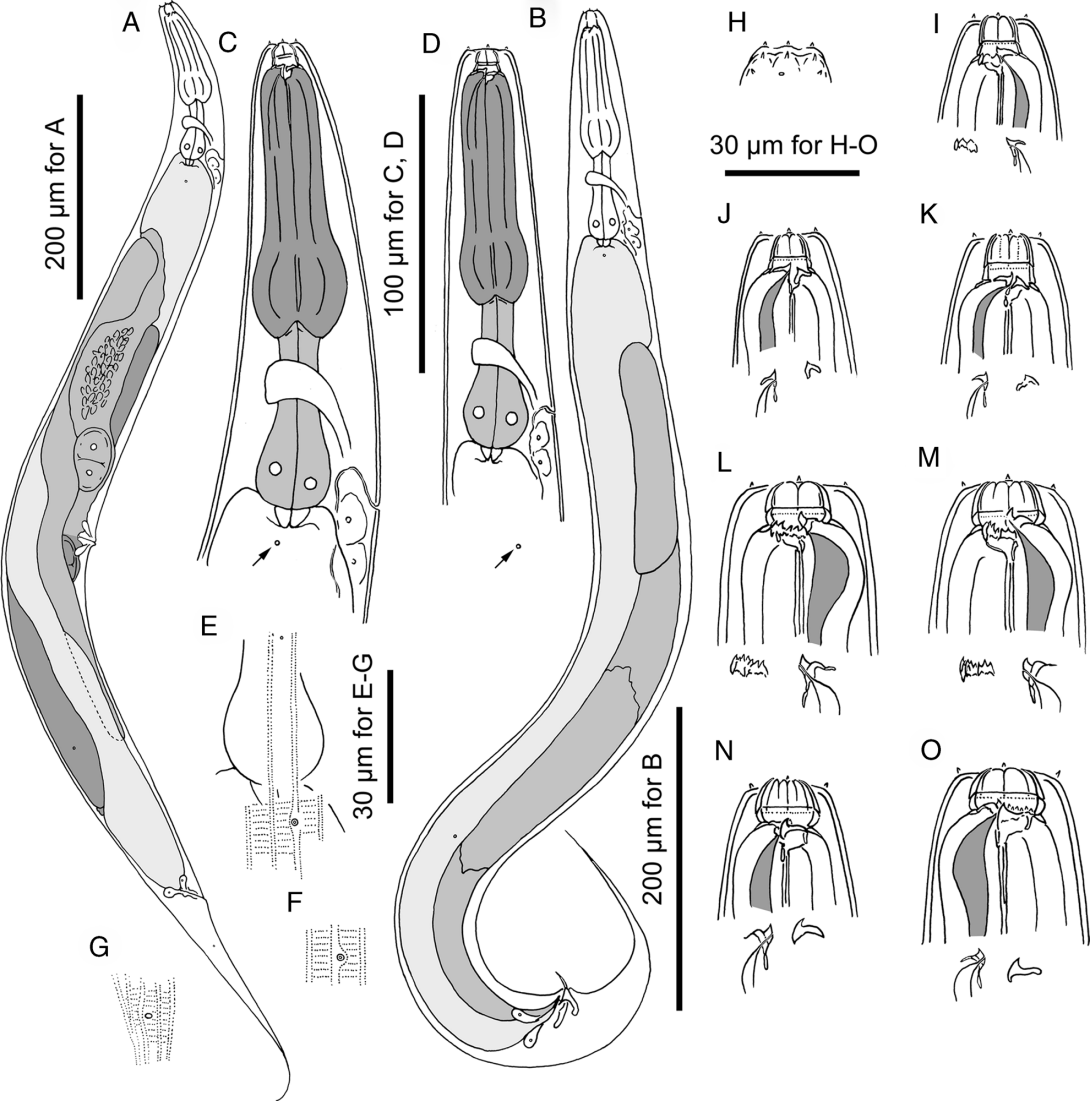
Adults of *Pristionchus paulseni* sp. n. A: female in right lateral view; B: male in right lateral view; C, D: anterior region of stenostomatous female (C) and male (D) in right lateral view showing the variation in body size and deirid position (arrow); E: body surface of female showing the relative position of deirid and lateral gland; F: postdeirid of female in left lateral view; G: phasmid opening of female in right lateral view; H: surface of male lip region in right lateral view; I: stomatal region of stenostomatal male in left lateral view; J, K: stomatal region of stenostomatal female in right lateral view showing the variation in cheilostomatal plates; L, M: stomatal region of eurystomatal female in left lateral view showing the variation in cheilostomatal plates; N: stomatal region of eurystomatal male in right lateral view showing partially split cheilostomatal plates; O: stomatal region of eurystomatal female in right lateral view.

**Figure 2:**
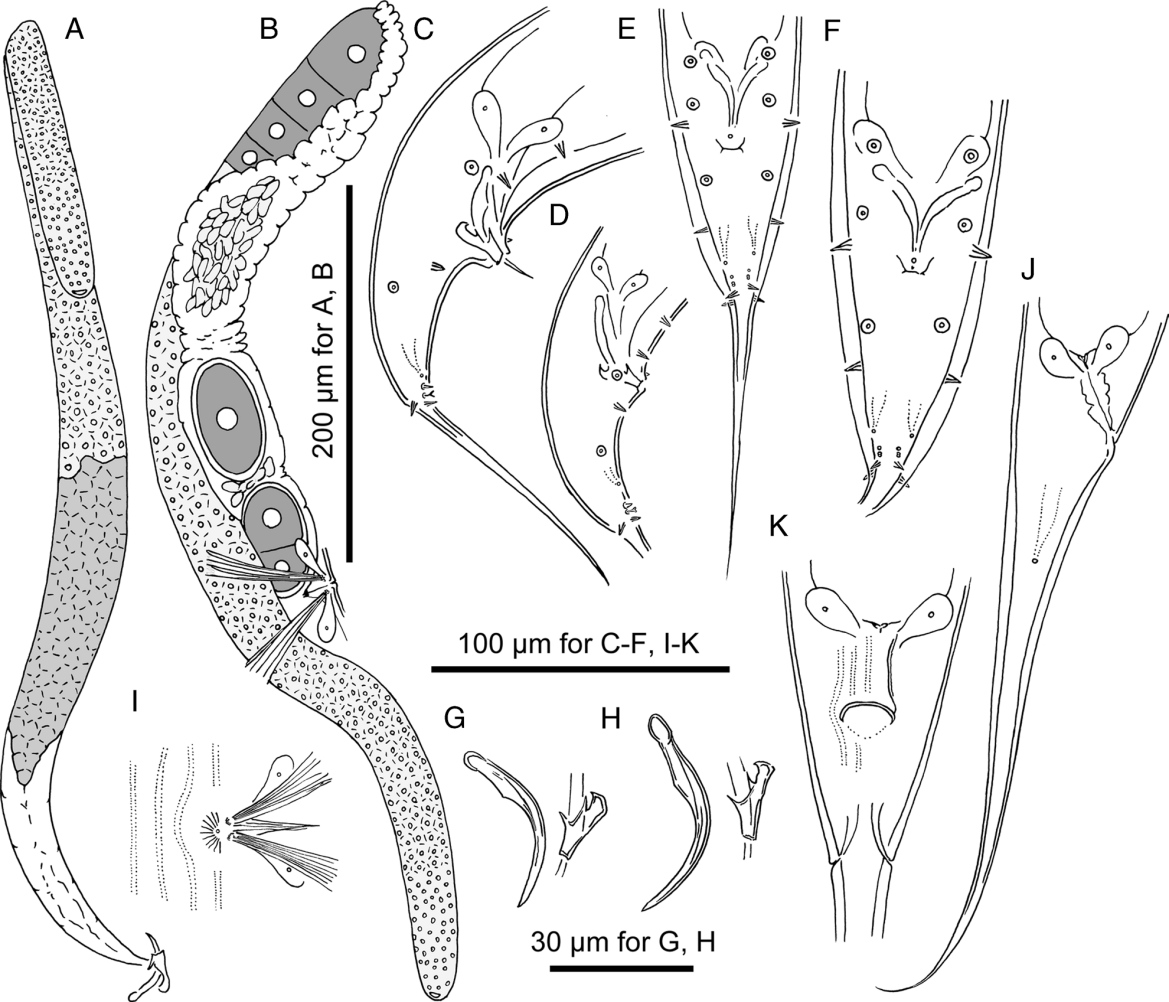
Adults of *Pristionchus paulseni* sp. n. A: male gonadal system in right lateral view; B; female anterior gonadal system in right lateral view; C, D: male tail region in right lateral view showing the variation in body size; E, F: male tail region in ventral view showing the variation in body size; G, H: spicule and gubernaculum in left lateral view showing the variation in body size; I: female vulval region in ventral view; J: female tail region in right lateral view; K: female anal region in ventral view.

Pristionchus paulseni* sp. n. (Figs 1, 2; Table 1)

**Table 1:** Morpometrics *P. paulseni* sp. n. and *P. yamagatae* sp. n.

	*P. paulseni* RS5918		*P. yamagatae* RS5964	
Character	stenostomatous male	stenostomatous female	stenostomatous male	stenostomatous female
n	10	10	10	10
L	1208±95.4 (990–1315)	1560±230 (1222–1895)	848±42.2 (796–916)	1162±121.9 (937–1385)
L’	1032±92.5 (813–1138)	1298±209.6 (978–1560)	681±34.7 (616–724)	908±103.7 (717–1096)
a	14±1,3 (12–16)	14±0.6 (13–15)	14±1.4 (13–18)	14±1.2 (12–17)
b	7.2±0.8 (5.8–9.0)	8.7±1.0 (7.3–10.1)	5.8±0.3 (5.3–6.2)	6.8±0.6 (5.8–7.8)
c	6.9±0.8 (5.6–8.3)	6.0±0.6 (5.0–6.9)	5.1±0.4 (4.4–5.9)	4.6±0.4 (4.1–5.3)
c’	4.0±0.6 (3.4–4.9)	6.0±0.5 (5.3–6.8)	5.1±0.6 (3.9–6)	7.3±0.7 (6.1–8.2)
T or V	58±2.9 (53–62)	47±0.9 (45–48)	51±3.3 (45–54)	45±1.3 (44–48)
Maximum body diam.	86±4.7 (75–91)	114±19.3 (84–138)	60±6.4 (47–70)	84±9.6 (68–98)
Stoma length	13.0±0.5 (12.0–13.8)	14.4±0.4 (13.9–15.0)	11.5±0.7 (10–12.2)	13.3±0.5 (12.3–14)
Stoma diam.	5.7±0.5 (5.0–6.8)	6.8±0.4 (6.2–7.5)	6±0.5 (5.4–6.7)	7.4±0.5 (6.7–8.3)
Pharynx length (head to base of pharynx)	156±13.8 (122–168)	169±7.2 (158–178)	135±5.4 (124–143)	156±7.3 (146–170)
Anterior pharynx (pro- + metacorpus)	96±8.3 (75–105)	106±2.9 (102–110)	83±3.9 (77–88)	98±4.2 (93–106)
Posterior pharynx (isthmus + basal bulb)	61±6.1 (47–68)	64±4.8 (56–69)	52±2.2 (47–55)	58±3.9 (52–64)
Ant/total pharynx %	61±1.3 (59–63)	62±1.6 (60–65)	63±4.2 (59–74)	63±1.2 (61–65)
Median bulb diam.	28±2.1 (25–31)	34±2.8 (30–36)	22±0.7 (21–23)	30±3.8 (22–36)
Terminal bulb diam.	26±1.8 (23–28)	30±3.9 (25–34)	21±1.3 (18–22)	27±3.7 (20–33)
Testis length	698±69.9 (532–787)	–	433±37.9 (372–475)	–
Ant. end to vulva	–	730±101.3 (592–860)	–	527±48.8 (446–610)
Vulva to anus distance	–	570±103.2 (424–710)	–	380±52.7 (281–470)
Cloacal or anal body diam.	44±3.4 (36–48)	44±4.3 (37–50)	33±3.6 (28–40)	35±1.9 (32–38)
Tail length	176±19.3 (155–220)	262±69.9 (235–335)	167±17.5 (140–192)	255±27.1 (212–289)
Spicule length (curve)	51±2.6 (47–54)	–	43±1.2 (41–45)	–
Spicule length (chord)	41±2.5 (36–44)	–	36±0.8 (34–37)	–
Gubernaculum length	14±1.3 (12–16)	–	14±1.4 (13–18)	–

*MH dedicates the species name to Dr. MJ Paulsen of Lincoln, Nebraska, entomologist expert, friend, long-term expedition fellow and secret twin brother.

### Measurements

Summarized in Table 1.

### Description of species specific characters

### Stenostomatous form

The anterior end of cheilostomatal plates is slightly elongated to form a small rounded flap in most cases. However, in a few cases, the anterior end splits into two tips having two small flaps, i.e., regardless of the number of plates (=6) resulting 6 to 12 flaps. Metastegostom bears a conspicuous and movable triangular or flint-shaped dorsal tooth with strongly sclerotized surface, giving an appearance of an inverted V-shape in light microscopy in lateral view; a pointed distal end, often curved to direct anteriorly; a pointed left subventral ridge with three minute adventitious denticles on a plate; and a pointed right subventral ridge, often with a distinct distal adventitious denticle.

### Eurystomatous form

Cheilostomatal plates are very thick. Anterior 1/3 to 1/2 sometimes split into two small tips. The anterior end of each plate is rounded and elongated to project from stomatal opening, forming a small flap; thus, the stoma has 6 to 12 flaps (=tips), regardless of the number of plates (=6). Each cheilostomatal plate is slightly inclined inwards, giving an appearance that the whole stoma is narrowing anteriorly. Gymnostom with very thick cuticle is present, forming a short, ring-like tube being thicker posteriorly and whole gymnostomatal ring widening anteriorly. The anterior end of gymnostom is internally overlapping the posterior end of cheilostomatal plates. Thus, the cheilo- and gymnostomatal regions form a barrel shape in lateral view. Metastegostom bears large claw-like dorsal tooth; large claw-like right subventral tooth; ridge of left subventral denticles or cusps, varying in number and size, i.e., three large cusps, the tip of these cusps is usually split into three or more small tips, sometimes forming serrated plate. The dorsal and right subventral teeth are movable. No movement is observed for left subventral denticles.

### Arrangement of genital papillae and phasmid

The paired papillae and the phasmid are arranged as <v1, v2, v3d, co, v4, ad, ph, (v5, v6, v7), pd>, where v1 is located at *ca* 1 CBD anterior to co; v2 and v3d are close to each other, but not overlapping, and v2 is located at less than 1/2 CBD anterior to co; v3d is at the midway between v2 and co; v4 *ca* 1/2 CBD is posterior to co; laterally located ad is at the midway between CO and the root of tail spike; ph is subventrally located in between ad and v5, but closer to v5; ventral v5 to v7 is forming triplet just anterior to the root of tail spike; and subdorsal pd is overlapping or slightly posterior to v7.

### Diagnosis and relationship

The species-specific (diagnostic) characters are described above. The new species is molecularly characterized by the barcoding sequence (NCBI accession number MH114982). In addition, the new species is typologically distinctive from many other *Pristionchus* spp with its barrel-shaped stoma in the eurystomatous form, i.e., the eurystomatous stoma of the *P. pacificus*, *P. maupasi* and *P. lhertieri*-groups has wide tube shapes, and the *elegans*-group has a tube-shaped stoma with membranous cheilostomatal plates (summarized in Ragsdale et al., 2015). A similar stomatal shape has been reported in the eury- and megastomatous forms of the *Pristionchus triformis* group (*P. triformis*, *P. hoplostomus* and *P. fukushimae*), and in the eurystomatous form of *P. fissidentatus* (Kanzaki et al., 2012a, b; Ragsdale et al., 2013). Although the new species share the partially split cheilostomatous plates with the megastomatous form of *P. fukushimae* and *P. hoplostomus* (and *P. yamagatae* n. sp., see below), *P. paulseni* sp. n. is distinguished from the *P. triformis* group by its anterior end of the gymnostom and pro-mesostegostom, and by the arrangement of the genital papillae, i.e., the most anterior sublaterally directed papillae are the third pair (v3d) *vs.* the second pair (v2d) (Ragsdale et al., 2013). In addition, *P. paulseni* sp. n. is distinguished from *P. triformis* by the reproductive mode, gonochoristic *vs*. hermaphroditic (Ragsdale et al., 2013). *P. paulseni* sp. n. is morphologically and phylogenetically closest to *P. fissidentatus*, but it is distinguished from *P. fissidentatus* by the cheilostomatous plates in both stenostomatous and eurystomatous forms, the arrangement of male genital papillae, especially the anterior four pairs, at *ca* 1 CBD anterior to co; v2 and v3d close to each other, but not overlapping, and v2 located at less than 1/2 CBD anterior to co; v3d at the midway between v2 and co; v4 *ca* 1/2 CBD posterior to co vs. v1 at 1.5 to 2 CBD anterior to co, and adcloacal v2 to v4 are gathered within 1/2 CBD from co, and finally the reproductive mode (Kanzaki et al., 2012a).

### Type host (carrier) and locality

The culture from which the type specimens were obtained was originally isolated by M. Herrmann from an adult *Lucanus dybowski taiwanus* collected at Pilu Sacred tree, Taroko National Park Taiwan in May 2017.

### Type material, type strain, and nomenclatural registration

One slide with holotype male and two slides, each with paratypes one male and one female, 28533-28535, were deposited in the University of California Riverside Nematode Collection (UCRNC), CA. Two slides, each with paratypes one male and one female (SMNHType-8991 and 8992) were deposited in the Swedish Natural History Museum, Stockholm, Sweden. Two slides, each with paratypes one male and one female (SMNK-NEMA-T-0145 and 0146) were deposited in the Natural History Museum Karlsruhe, Germany. The strain is available as living culture and as frozen stocks under culture code RS5918 in the Department of Evolutionary Biology, Max Planck Institute (MPI) for Developmental Biology, Tübingen, Germany, and it can be provided to other researchers upon request. The new species binomial has been registered in the ZooBank database (zoobank.org) under the identifier [urn:lsid:zoobank.org:act:6E8BF33F-6F9E-4A6E-99C2-5576242EC26E].

**Figure 3:**
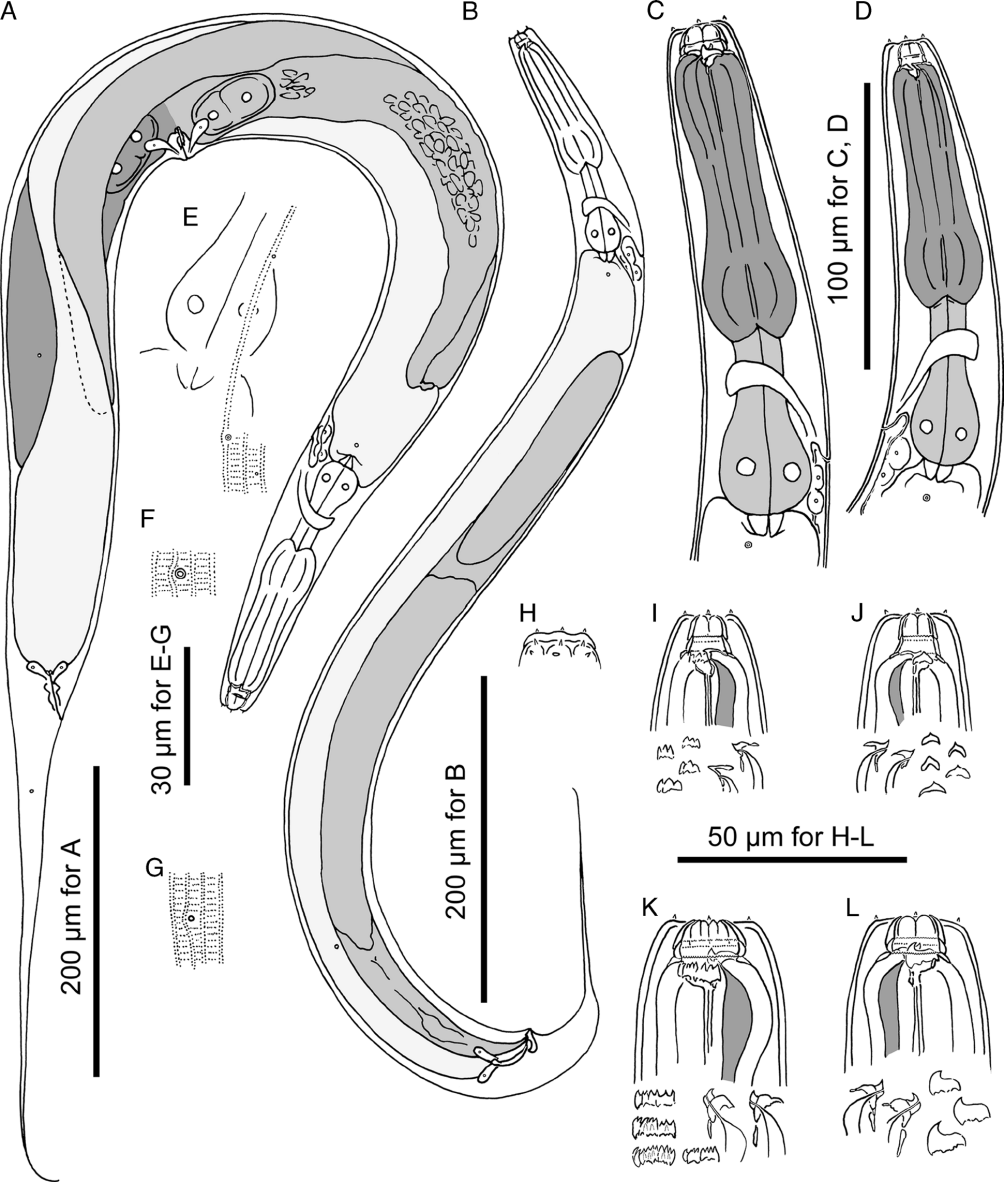
Adults of *Pristionchus yamagatae* sp. n. A: female in right lateral view; B: male in right lateral view; C, D: anterior region of eurystomatous (C) and stenostomatous (D) females in right and left lateral views, respectively; E: body surface of female showing the relative position of deirid and lateral gland; F: excretory pore opening in ventral view; G: phasmid opening of female in right lateral view; H: surface of male lip region in right lateral view; I: stomatal region of stenostomatal female in left lateral view; J: stomatal region of stenostomatal female in right lateral view; K: stomatal region of eurystomatal female in left lateral view showing partially split cheilostomatal plates; L: stomatal region of eurystomatal female in right lateral view.

**Figure 4:**
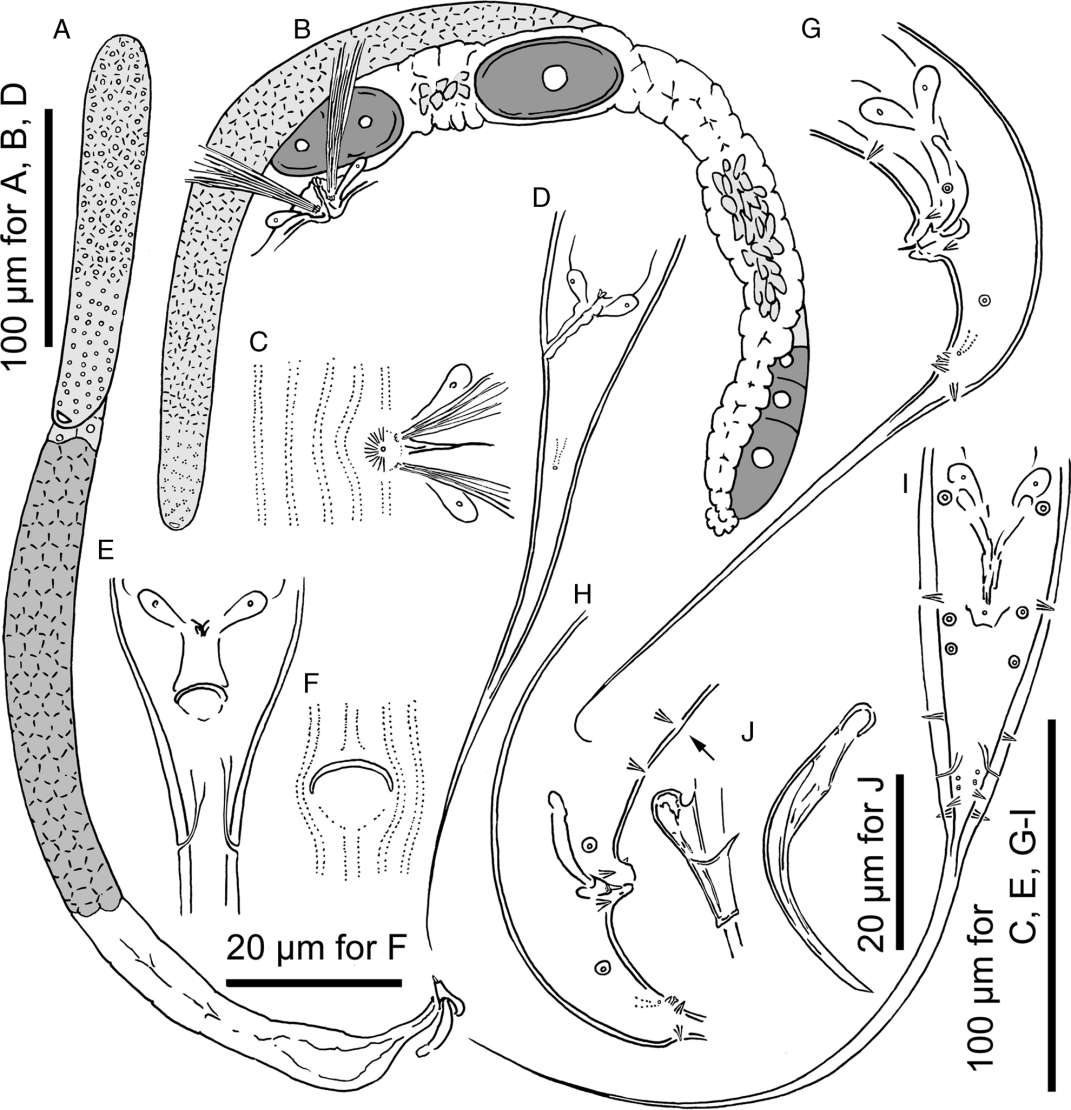
Adults of *Pristionchus yamagatae* sp. n. A: male gonadal system in right lateral view; B: female anterior gonadal system in right lateral view; C: female vulval region in ventral view; D: female tail region in left lateral view; E: female anal region in ventral view; F: surface striation of female anal region in ventral view; G: male tail region in left lateral view; H: male tail region in right lateral view showing the presence of extra v0 papilla (arrow); I: male tail region in ventral view; J: spicule and gubernaculum in right lateral view.

Pristionchus yamagatae* sp. n. (Figs 3, 4; Table 1).

*The species name is derived from the type locality of the nematode, Yuza, Yamagata Prefecture, Japan.

### Measurements

Summarized in Table 1

### Stenostomatous form

The anterior ends of gymnostomatal and pro-mesostegostomatal rings are weakly serrated, but they are difficult to observe under light microscopy. Metastegostom bears conspicuous and movable triangular or flint-shaped dorsal tooth with strongly sclerotized surface, giving an appearance of an inverted V-shape in light microscopy in lateral view; a pointed distal end often curved to direct anteriorly; a pointed left subventral ridge with three minute adventitious denticles on a plate; bluntly or sharply pointed right subventral ridge, often with a distinct distal adventitious denticle.

### Eurystomatous form

Cheilostomatal plates are very thick. The anterior end of each plate is rounded and elongated to project from stomatal opening, forming a small flap. In the anterior part, only the tip to anterior half of each cheilostomatal plate sometimes splits into two small flaps, i.e., stomatal opening bearing 6 to 12 flaps (= tips), regardless of the number of plates (=6). Each cheilostomatal plate is slightly inclined inwards, giving an appearance that the whole stoma is narrowing anteriorly. Gymnostom with a very thick cuticle is present, forming a short, ring-like tube being thicker posteriorly, and whole gymnostomatal ring widening anteriorly. The anterior end of gymnostom is weakly serrated and it internally overlaps the posterior end of cheilostomatal plates. Thus, the cheilo and gymnostomatal regions form a barrel shape in lateral view. The anterior edge of pro-mesostegostom is clearly serrated. Metastegostom bears a large claw-like dorsal tooth; a large claw-like right subventral tooth; ridge of left subventral denticles or cusps, varying in number and size, i.e., three large cusps, the tip of these cusps is usually split into three or more small tips, sometimes forming serrated plate. The dorsal and right subventral teeth are movable. No movement is observed for left subventral denticles.

### Arrangement of genital papillae and phasmid

The paired papillae and the phasmid are arranged as <v1, v2d, v3, co, v4, ad, ph, (v5, v6, v7), pd>, where v1 is located at *ca* 1 CBD or slightly more anterior to co; v2d and v3 close to each other, but not overlapping, and v2 is located at *ca* 1/3 CBD anterior to co; v3 at the midway between v2d and co; v4 is less than 1/3 CBD posterior to co; thus, the distance between v2d and v4 is less than 1 CBD; laterally located ad is at the midway between co and the root of tail spike; ph is subventrally located just anterior to or overlapping with v5; ventral v5 to v7 is forming triplet just anterior to the root of tail spike; and subdorsal pd is overlapping or slightly posterior to v7. In addition to regular nine pairs of papillae, a few individuals have an extra pair of subventral papillae anterior to v1 (= v0).

### Diagnosis and relationship

The diagnostic characters are described above. This new species is molecularly characterized by the barcoding sequence (NCBI accession number MH114983). Also, the new species is typologically distinctive from many other *Pristionchus* spp. with its barrel-shaped stoma with weakly serrated anterior end of gymnostomatal ring and clearly serrated anterior end of pro-mesostegostomatal ring. These specific characters suggest that *P. yamagatae* n. sp. belongs to the *P. triformis* group including *P. triformis*, *P. hoplostomus* and *P. fukushimae*. These three species share the stomatal structure with the new species (Ragsdale et al., 2013, 2015). However, *P. yamagatae* n. sp. is readily distinguished from *P. triformis* by its reproductive mode, gonochoristic vs hermaphroditic, the cheilostomatal plates in the stenostomatous and the eurystomatous forms, the arrangement of male genital papillae, v2d and v3 being close to each other vs clearly separated, and relative position of phasmid, located just anterior to or overlapping with v5 vs being located midway between ad and pd paired papillae, or more anteriorly, and clearly separated from v5 (Ragsdale et al., 2013). The new species is distinguished from *P. hoplostomus* by the cheilostomatal plates in the stenostomatous form, i.e., the twelve-plated stenostomatous form was not found, the arrangement of genital papillae, the level of v4 being clearly apart from cloacal opening vs close to cloacal opening, and the relative position of phasmid, located just anterior to or overlapping with v5 vs being located midway between ad and pd paired papillae, or more anteriorly, and clearly separated from v5 (Ragsdale et al., 2013). *P. yamagatae* sp. n. is also distinguished from *P. fukushimae* by the cheilostomatal plates in the stenostomatous form, and the relative position of phasmid, located just anterior to or overlapping with v5 vs being located midway between ad and pd paired papillae, or more anteriorly, and clearly separated from v5 (Ragsdale et al., 2013). In addition to these typological characters, *P. yamagatae* sp. n. is distinguished from its close relatives by its molecular phylogenetic status and mating experiments.

### Type host (carrier) and locality

The culture from which the type specimens were obtained was originally isolated by N. Kanzaki from an adult *Holotrichia kyotoensis* collected at a Mamurogawa, Yamagata, Japan in July 2017.

### Type material, type strain, and nomenclatural registration

One slide with holotype male and two slides, each with paratypes one male and one female, 28536–28538, were deposited in the University of California Riverside Nematode Collection (UCRNC), CA. Two slides, each with paratypes one male and one female (SMNHType-8993 and 8994), were deposited in the Swedish Natural History Museum, Stockholm, Sweden. Two slides, each with paratypes one male and one female (SMNK-NEMA-T-0147 and 0148), were deposited in the Natural History Museum Karlsruhe, Germany. The strain is available as living cultures and as frozen stocks under culture code RS5964 in the Department of Evolutionary Biology, MPI for Developmental Biology, Tübingen, Germany, and it can be provided to other researchers upon request. The new species binomial has been registered in the ZooBank database (zoobank.org) under the identifier [urn:lsid:zoobank.org:act:2A1FAEB0-0D51-4563-A043-34B1515206A4].

### Molecular characterization and phylogenetic analysis

**Figure 5:**
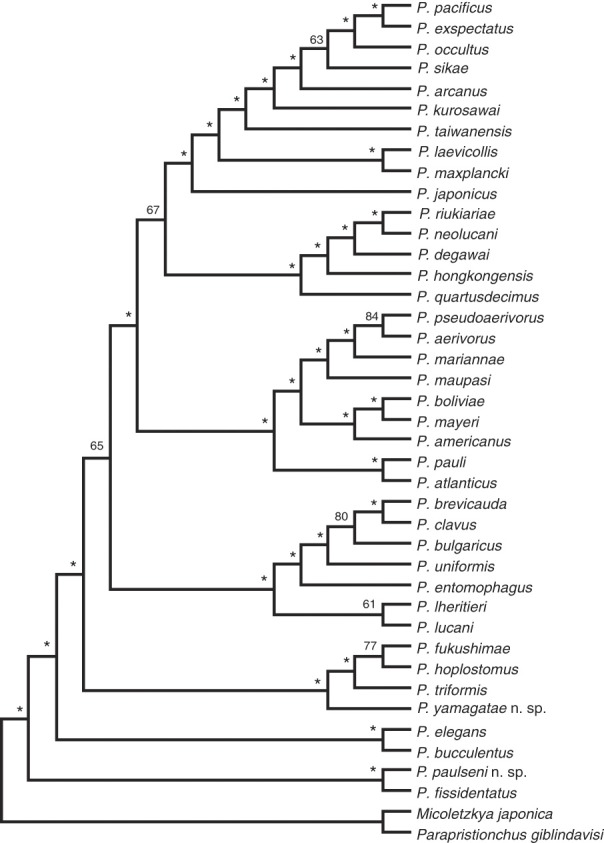
Phylogenetic relationships of the *Pristionchus* genus. The schematic phylogeny shows the relationship between all species of the *Pristionchus* genus as inferred from transcriptome data (Rödelsperger et al., 2018), including those described in the two accompanying manuscripts (Yoshida et al., 2018; Kanzaki et al., 2018).

The phylogenetic positions of the two novel species were determined by phylogenetic analysis of transcriptomes from all cultivable *Pristionchus* species (Rödelsperger et al. 2018). The resulting phylogeny of the genus, as shown in Figure 5, places *P. yamagatae* sp. n. into the subclade consisting of *P. triformis*, *P. hoplostomus*, and *P. fukushimae*. More importantly, this phylogeny positions *P. paulseni* sp. n. as sister species to the hermaphroditic *P. fissidentatus*. Thus, except for *P. mayeri* and *P. boliviae*, all hermaphroditic species in the *Pristionchus* genus have at least one relatively close gonochoristic sister species.

## Discussion

In this study, we have described two new *Pristionchus* species with the particular importance of *P. paulseni* sp. n. for two major reasons. First, *P. paulseni* sp. n. is most closely related to the hermaphrodite *P. fissidentatus*, a species that formerly had no known direct gonochoristic sister species. The comparison between *Pristionchus* hermaphrodites and their gonochoristic sister species allows important experimental inroads from mating-type-related traits such as longevity to comparative genomics (Weadick and Sommer, 2016; Rödelsperger et al., 2018). Therefore, the identification of *P. paulseni* sp. n. fills an important gap for the taxonomy and phylogeny of *Pristionchus* species and represents the fifth hermaphroditic–gonochoristic species pair in this genus.

Also, the species pair *P. paulseni* sp. n. and *P. fissidentatus* is the sister group to all described scarab beetle-associated *Pristionchus* species. Therefore, the characters of these species will be important for character polarization in the complete genus. For example, recent studies have started to investigate genome architecture across *Pristionchus* nematodes and basal gonochoristic species will be of critical importance (Rödelsperger et al., 2018). Also, the functional investigation of mouth-form plasticity in *P. pacificus* involves comparative studies including multiple *P. pacificus* strains and other *Pristionchus* species (for recent review see, Sommer et al., 2017). Ultimately, deep taxon sampling of basal species will result in a stronger support of character evolution. In the context of *Pristionchus* mouth-form plasticity, the fact of *P. paulseni* sp. n. and *P. fissidentatus* being dimorphic provides a strong support for plasticity being ancestral, whereas the single morphs known from *P. elegans* and *P. bucculentus* most likely represent examples of secondary losses. Thus, deep taxon sampling increases the robustness of *Pristionchus* phylogeny and character polarizations.
